# Excessive Thyroid Hormone Signaling Induces Photoreceptor Degeneration in Mice

**DOI:** 10.1523/ENEURO.0058-23.2023

**Published:** 2023-09-01

**Authors:** Hongwei Ma, Fan Yang, Lilliana R. York, Shujuan Li, Xi-Qin Ding

**Affiliations:** Department of Cell Biology, University of Oklahoma Health Sciences Center, Oklahoma City, Oklahoma 73104

**Keywords:** cone, mice, photoreceptor, retina, rod, thyroid hormone

## Abstract

Rod and cone photoreceptors degenerate in inherited and age-related retinal degenerative diseases, ultimately leading to loss of vision. Thyroid hormone (TH) signaling regulates cell proliferation, differentiation, and metabolism. Recent studies have shown a link between TH signaling and retinal degeneration. This work investigates the effects of excessive TH signaling on photoreceptor function and survival in mice. C57BL/6, *Thra1*^−/−^, *Thrb2*^−/−^, *Thrb*^−/−^, and the cone dominant *Nrl*^−/−^ mice received triiodothyronine (T3) treatment (5–20 μg/ml in drinking water) for 30 d, followed by evaluations of retinal function, photoreceptor survival/death, and retinal stress/damage. Treatment with T3 reduced light responses of rods and cones by 50–60%, compared with untreated controls. Outer nuclear layer thickness and cone density were reduced by ∼18% and 75%, respectively, after T3 treatment. Retinal sections prepared from T3-treated mice showed significantly increased numbers of TUNEL-positive, p-γH2AX-positive, and 8-OHdG-positive cells, and activation of Müller glial cells. Gene expression analysis revealed upregulation of the genes involved in oxidative stress, necroptosis, and inflammation after T3 treatment. Deletion of *Thra1* prevented T3-induced degeneration of rods but not cones, whereas deletion of *Thrb2* preserved both rods and cones. Treatment with an antioxidant partially preserved photoreceptors and reduced retinal stress responses. This study demonstrates that excessive TH signaling induces oxidative stress/damage and necroptosis, induces photoreceptor degeneration, and impairs retinal function. The findings provide insights into the role of TH signaling in retinal degeneration and support the view of targeting TH signaling for photoreceptor protection.

## Significance Statement

Thyroid hormone (TH) signaling regulates cell growth, differentiation, and metabolic homeostasis. In the retina, TH signaling has been linked to the progression of retinal degeneration. Inhibition of TH signaling protects photoreceptors from cell death in mouse models of retinal degeneration. The degenerating retinas show increased expression/activity of TH signaling components. Moreover, clinical studies have shown an association between elevated TH level in the circulation/hyperthyroidism and increased incidence of retinal degeneration/age-related macular degeneration. This work demonstrates the detrimental effects of excessive TH signaling in mouse photoreceptors. The findings provide insights into the role of TH signaling in retinal degeneration. Strategies targeting TH signaling may help reduce or slow down retinal/photoreceptor degeneration.

## Introduction

In mammals, the thyroid gland predominantly produces the thyroid hormone (TH) prohormone thyroxine (T4; ∼90%), along with a small amount of the bioactive hormone triiodothyronine (T3; 5–10%). The conversion of T4 to T3 in cells is primarily catalyzed by the type 2 iodothyronine deiodinases (Dio2). Intracellular T4 and T3 are degraded by the type 3 iodothyronine deiodinases (Dio3). TH signaling regulates cell proliferation, differentiation, and metabolism (Forrest et al., 2002; Cheng et al., 2010; Brent, 2012), and has also been associated with cell death/survival. TH signaling is the main driving force in apoptotic tissue remodeling during anuran metamorphosis (Shi et al., 2001; Buchholz et al., 2004). TH signaling is associated with apoptosis of a variety of human cell lines, including lymphocytes (Mihara et al., 1999), breast cancer cells (Sar et al., 2011), HeLa cells (Yamada-Okabe et al., 2003), and pituitary tumor cells (Chiloeches et al., 2008). Excessive TH signaling has been shown to induce auditory defects/cochlear degeneration (Ng et al., 2009b) and cerebellum degeneration (Peeters et al., 2013) in mice. More importantly, TH signaling has been linked to neurodegenerative conditions in humans, including Alzheimer’s disease (Kalmijn et al., 2000; Ceresini et al., 2009) and age-related macular degeneration (AMD; Age-Related Eye Disease Study Research Group, 2000; Chaker et al., 2015; Gopinath et al., 2016; Chatziralli et al., 2017; Lin et al., 2018; Abdelkader and Abass, 2019; Farvardin et al., 2021; Xu et al., 2021; Hung et al., 2022; Li et al., 2022).

Rod and cone photoreceptors play a central role in vision. Rods and cones degenerate in a variety of pathologic conditions, including inherited retinal degenerative diseases such as retinitis pigmentosa, Leber congenital amaurosis (LCA), and cone–rod dystrophies, and age-related retinal degeneration diseases such as AMD. Inherited retinal degenerative diseases affect ∼1 in 3000 individuals worldwide, and AMD is the leading cause of blindness among the aged population. The highly heterogeneous nature of these diseases is a challenge when developing therapeutic strategies, and there is currently no treatment for retinal degeneration. Nevertheless, the degenerating photoreceptors show some common cellular disorder features despite a high genetic heterogeneity, including oxidative damage (Shen et al., 2005; Komeima et al., 2006), inflammatory lesions (Cruz-Guilloty et al., 2013; Huang et al., 2018; Allocca et al., 2019), apoptosis (Dunaief et al., 2002; Sanges et al., 2006), and necroptosis (Viringipurampeer et al., 2014; Huang et al., 2018; Allocca et al., 2019). These features suggest a likelihood of some common mechanisms governing the cellular degeneration/death process and offer the possibility of targeting common cellular survival and death regulators/pathways to reduce photoreceptor death, regardless of the genetic origins of the diseases.

In the retina, TH signaling is well known for its regulation in cone opsin expression/patterning and cone development. It suppresses short wave-sensitive opsin 1 expression, induces medium wave-sensitive opsin 1 (M-opsin) expression (Roberts et al., 2006; Glaschke et al., 2011), and controls the dorsal–ventral gradient expression of cone opsin (Roberts et al., 2006). TH signaling has also been linked to photoreceptor viability/cone degeneration. Suppression of TH signaling by antithyroid treatment, inhibition of Dio2, overexpression of Dio3, or deletion of TH receptor reduces cone degeneration in mouse models of LCA, cone dystrophy/achromatopsia (Ma et al., 2014; Yang et al., 2016), and a chemically induced mouse model of AMD (Ma et al., 2020, 2022).

The present work examined the effects of excessive TH signaling on photoreceptor viability and retinal function in mice. Treatment with T3 induced cone/rod degeneration and reduced retinal light responses. Deletion of *Thra1* prevented T3-induced degeneration of rods but not cones, whereas deletion of *Thrb2* preserved both rods and cones. Biochemical/gene expression analysis showed that TH-induced photoreceptor degeneration likely involves multiple cellular mechanisms, including oxidative stress, necroptosis, and inflammation. This work demonstrates the detrimental effects of excessive TH signaling in mouse photoreceptors and supports the view that the inhibition of TH signaling might be a valuable strategy for photoreceptor protection.

## Materials and Methods

### Mice and reagents

C57BL/6J and *Thra1*^−/−^ (Wikström et al., 1998) mouse lines were obtained from The Jackson Laboratory, *Thrb2*^−/−^ (Ng et al., 2001), *Thrb*^−/−^ (lacking both of the isoform splicing variants Thrb1 and Thrb2; Forrest et al., 1996) mouse lines were provided by Douglas Forrest (National Institute of Diabetes and Digestive and Kidney Diseases, NIH), and the *Nrl*^−/−^ (Mears et al., 2001) mouse line was provided by Anand Swaroop (Neurobiology Neurodegeneration & Repair Laboratory, National Eye Institute, NIH). Mice were maintained under a cyclic light condition (12 h light/dark cycle). Cage illumination was 7 foot-candle during the light cycle. All animal maintenance and experiments were approved by the local Institutional Animal Care and Use Committee (University of Oklahoma Health Sciences Center) and conformed to the Guidelines on the Care and Use of Animals of the Society for Neuroscience and the Association for Research in Vision and Ophthalmology. Mice of either sex were used in the experiments and randomly assigned within a litter for the drug treatment or vehicle/untreated experiments. Antibodies and reagents used in the experiments are listed in Table 1.

**Table 1:** Antibodies/reagents and conditions used in this study

Antibodies/reagent	Vendor	Catalog #	Dilutions used in IF or IB
3,3′,5-Triiodo-l-thyronine	Millipore Sigma	D9542	
DAPI	Millipore Sigma	T2877	1:2000 (IF)
Biotinylated PNA	Vector Laboratories	B-1075	1:200 (IF)
Anti-8-OHdG	Santa Cruz Biotechnology	Sc-393871	1:200 (IF)
Anti-γH2AX (p-Ser139)	Novus	NB100-2280	1:200 (IF)
Anti-GFAP	DAKO	Z0334	1:500 (IF)
Anti-β-actin	Abcam	Ab6276	1:2000 (IF)
Anti-p-Ripk3	Abcam	Ab195117	1:500 (IB), 1:200 (IF)
Anti-p-Mlkl	Abcam	Ab196436	1:500 (IB), 1:200 (IF)
Alexa Fluor 555 goat anti-rabbit IgG	Thermo Fisher Scientific	A21428	1:500 (IF)
Streptavidin-Cy3	Thermo Fisher Scientific	SA1010	1:500 (IF)

DAPI, 4′,6′-Diamidino-2-phenylindole dihydrochloride. IF, Immunofluorescence labeling; IB, immunoblotting.

### Scotopic and photopic electroretinography recordings

Full-field electroretinography (ERG) recordings were conducted as described previously (Xu et al., 2012). Briefly, after overnight dark adaptation, mice were anesthetized by intraperitoneal injection of 85 mg/kg ketamine and 14 mg/kg xylazine. ERGs were recorded using the Espion Visual Electrophysiology System (Diagnosys) with the ColorDome Advanced Performance Ganzfeld Dome system (Diagnosys). Potentials were recorded using a gold-wire electrode to contact the corneal surface through a layer of 2.5% hypromellose (Gonak, Akorn Pharmaceuticals). For the assessment of scotopic responses, a stimulus intensity of 1.89 log cd · s m^−2^ was presented to dark-adapted dilated mouse eyes. To evaluate photopic responses, mice were adapted to a 1.48 log cd · s m^−2^ light for 7 min, and then a light intensity of 1.89 log cd · s m^−2^ was given. Responses were differentially amplified, averaged, and analyzed using Espion 100 software (Diagnosys).

### Eye preparation, immunofluorescence labeling, confocal microscopy, and morphometric analysis

Retinal whole mounts or cross sections were prepared for immunofluorescence labeling, as described previously (Ma et al., 2014). For retinal whole-mount preparations, eyes were enucleated, marked at the superior pole with a green dye, and fixed in 4% paraformaldehyde (PFA; Polysciences) for 30 min at room temperature, followed by removal of the cornea and lens. The eyes were then fixed in 4% PFA for 4–6 h at room temperature, retinas were isolated, and the superior portion was marked for orientation with a small cut. For retinal cross sections, mouse eyes were enucleated (the superior portion of the cornea was marked with green dye before enucleation) and fixed in Prefer (Anatech) for 25–30 min at room temperature before being transferred into 70% ethanol. Paraffin sections (5 μm thickness) passing vertically through the retina (along the vertical meridian passing through the optic nerve head) were prepared using a microtome (Leica Biosystems).

Immunofluorescence labeling was performed as described previously (Ma et al., 2014). Briefly, retinal whole mounts were blocked with HBSS containing 5% bovine serum albumin (BSA) and 0.5% Triton X-100 overnight at 4°C. Peanut-agglutinin (PNA) immunohistochemistry was performed using biotinylated PNA and then streptavidin-Cy3 at room temperature for 1 h. For immunofluorescence staining on sections, after deparaffin and rehydration steps, antigen retrieval was performed in 10 mm sodium citrate buffer, pH 6.0, in a 70°C water bath. Primary antibody incubation was performed overnight at 4°C. Table 1 shows dilutions of the antibodies used. Slides were mounted and coverslipped after fluorescence-conjugated secondary antibody incubation and wash steps. Immunofluorescence was imaged using a confocal laser-scanning microscope (model FV1000, Olympus) and FluoView imaging software (Olympus). Evaluation of glial fibrillary acidic protein (GFAP) fluorescence density was conducted as described previously (Ma et al., 2019). Briefly, images from the central, middle, and peripheral retinal regions were taken, and confocal images of 10 layers of each region were stacked with the *z*-stack function in the ImageJ software (https://imagej.nih.gov/ij/) to obtain a maximal immunofluorescence density. Fluorescence density levels of the immunolabeling in the center, middle, and peripheral regions were measured after the removal of the background, and the average fluorescence density from the three regions was used for statistical analysis. For retinal morphometric analysis, retinal cross sections stained with hematoxylin and eosin (H&E) were used to evaluate outer nuclear layer (ONL) thickness/rod survival, as described previously (Xu et al., 2012; Ma et al., 2014).

### Measurement of T3 in circulation

Serum T3 levels were analyzed using a mouse/rat T3 ELISA kit (catalog #T3043T-100, Calbiotech) with a total T3 detection limit at 0.25 ng/ml, as described previously (Ma et al., 2020). Briefly, 25 μl of serum samples and standards with different T3 concentrations were added into the assigned wells, the assays were performed by following the manufacturer instruction, and the absorbance of each well was read at 450 nm (SpectraMax 190 Microplate Spectrophotometer, Molecular Devices). The standard curve was generated by using a three-parameter exponential nonlinear regression in SigmaPlot software, and the sample T3 concentration was then calculated according to the three-parameter exponential equation.

### Terminal deoxynucleotidyl transferase-mediated biotinylated UTP nick end labeling

Terminal deoxynucleotidyl transferase-mediated biotinylated UTP nick end labeling (TUNEL) was performed on paraffin-embedded retinal sections, using an In Situ Cell-Death Fluorescein-Detection kit (catalog #11684795910, Millipore Sigma), as described previously (Ma et al., 2015). Immunofluorescence signals were imaged using a confocal laser-scanning microscope (model FV1000, Olympus). TUNEL-positive cells in the outer nuclear layer passing through the optic nerve were counted and averaged from at least three sections per eye, from three to four mice per condition.

### Drug treatment

T3 for drinking water was prepared as described previously (Hamidi et al., 2010). Ten milligrams of T3 (catalog #T2877, Sigma-Aldrich) were dissolved in 1.0 ml of 1.0N NaOH, followed by dilution with tap water for final working concentrations.

*N*-acetylcysteine (NAC; 200 mg/kg/d; catalog #A0737, Millipore Sigma), dissolved in saline (Schimel et al., 2011) and filtered with 0.45 μm filter, was administered to mice by intraperitoneal injection once daily.

### RNA isolation and quantitative real-time PCR

Total RNA preparation and reverse transcription were performed as described previously (Ma et al., 2013). The gene encoding the mouse hypoxanthine guanine phosphoribosyl transferase 1 (*Hprt1*) was included as an internal control. Table 2 shows the primers used. The quantitative real-time PCR (qRT-PCR) assays were performed using a real-time PCR detection system (iCycler, BIO-RAD), and the relative gene expression value was calculated based on the ΔΔCt method, as described previously (Ma et al., 2013).

**Table 2:** Primers used for qRT-PCR

Gene	Forward primer	Reverse primer
*Hprt1*	GCAAACTTTGCTTTCCCTGGTT	CAAGGGCATATCCAACAACA
*Dio3*	GTGGTCGGAGAAGGTGAAG	TGCACAAGAAATCTAAAAGCCAG
*Il1α*	TGCAGTCCATAACCCATGATC	ACAAACTTCTGCCTGACGAG
*Il1β*	ACGGACCCCAAAAGATGAAG	TTCTCCACAGCCACAATGAG
*Il6*	CAAAGCCAGAGTCCTTCAGAG	GTCCTTAGCCACTCCTTCTG
*Mlkl*	ACTGTGAACTTGGAACCCTG	TGCTGATGTTTCTGTGGAGTG
*Nlrp3*	CTCCAACCATTCTCTGACCAG	ACAGATTGAAGTAAGGCCGG
*Ripk1*	GGAAGGATAATCGTGGAGGC	AAGGAAGCCACACCAAGATC
*Ripk3*	TCTTTACTGAGACTCCCGGT	AGTTCCCAATCTGCACTTCAG
*Tnf1α*	CTTCTGTCTACTGAACTTCGGG	CAGGCTTGTCACTCGAATTTTG
*Tnfrsf1a*	CTCTGCTCTACGAATCACTCTG	CACAGCATACAGAATCGCAAG
*Tnfrsf9*	CCTGTGATAACTGTCAGCCTG	TCTTGAACCTGAAATAGCCTGC
*Tradd*	ACGAACTCACTAGTCTAGCAGAG	AATACCCCAACAGCCACC

### Retinal protein preparation, SDS-PAGE, and Western blot analysis

Retinal protein preparation, SDS-PAGE, and Western blot analysis were performed as described previously (Ma et al., 2015). Briefly, retinas were homogenized in homogenization buffer A [0.32 m sucrose, 20 mm HEPES, pH 7.4, and 3 mm EDTA containing protease and phosphatase inhibitors (catalog #04693159001 and #04906837001, respectively, Roche Life Science)], and homogenates were centrifuged at 3000 rpm for 10 min at 4°C. The resulting supernatant was then centrifuged at 13,000 rpm for 35 min at 4°C to separate cytosolic (supernatant) and membrane (pellet) fractions. The cytosolic fractions were used, and protein concentrations were determined by a protein assay kit from BIO-RAD. Retinal protein samples were then subjected to SDS-PAGE and transferred to PVDF membranes, which were subsequently blocked in 5% milk for 1 h at room temperature. Immunoblots were incubated with primary antibody overnight at 4°C. Table 1 shows dilutions of the antibodies used. After washing in Tris-buffered saline with 0.1% Tween 20, immunoblots were incubated with horseradish peroxidase-conjugated secondary antibody (1:20,000) for 1 h at room temperature. SuperSignal West Dura Extended Duration chemiluminescent substrate (catalog #34076, Thermo Fisher Scientific) was used to detect binding of the primary antibodies to their cognate antigens. Odyssey CLx Imager and software (LI-COR) were used for detection and densitometric analysis.

### Statistical analysis

The results are expressed as the mean ± SEM of the number of mice or the number of assays. Power analysis was performed to choose the sample size. The analysis indicates that a sample size of three to five mice per group for evaluations of retinal degeneration in the mouse retinas will provide at least 80% power (1-β) for a two-sided, two-sample *t* test at a 0.05 α level. One-way ANOVA was used for significance within sets of data, followed by Dunn’s multiple-comparisons test. An unpaired Student’s *t* test/Mann–Whitney test was used for differences between two groups of data. Data were analyzed using the nonparametric tests. Differences were considered statistically significant at *p *<* *0.05. Data were analyzed and graphed using GraphPad Prism software (GraphPad Software).

## Results

### Treatment with T3 impairs retinal function and induces rod and cone degeneration

**Figure 1.**
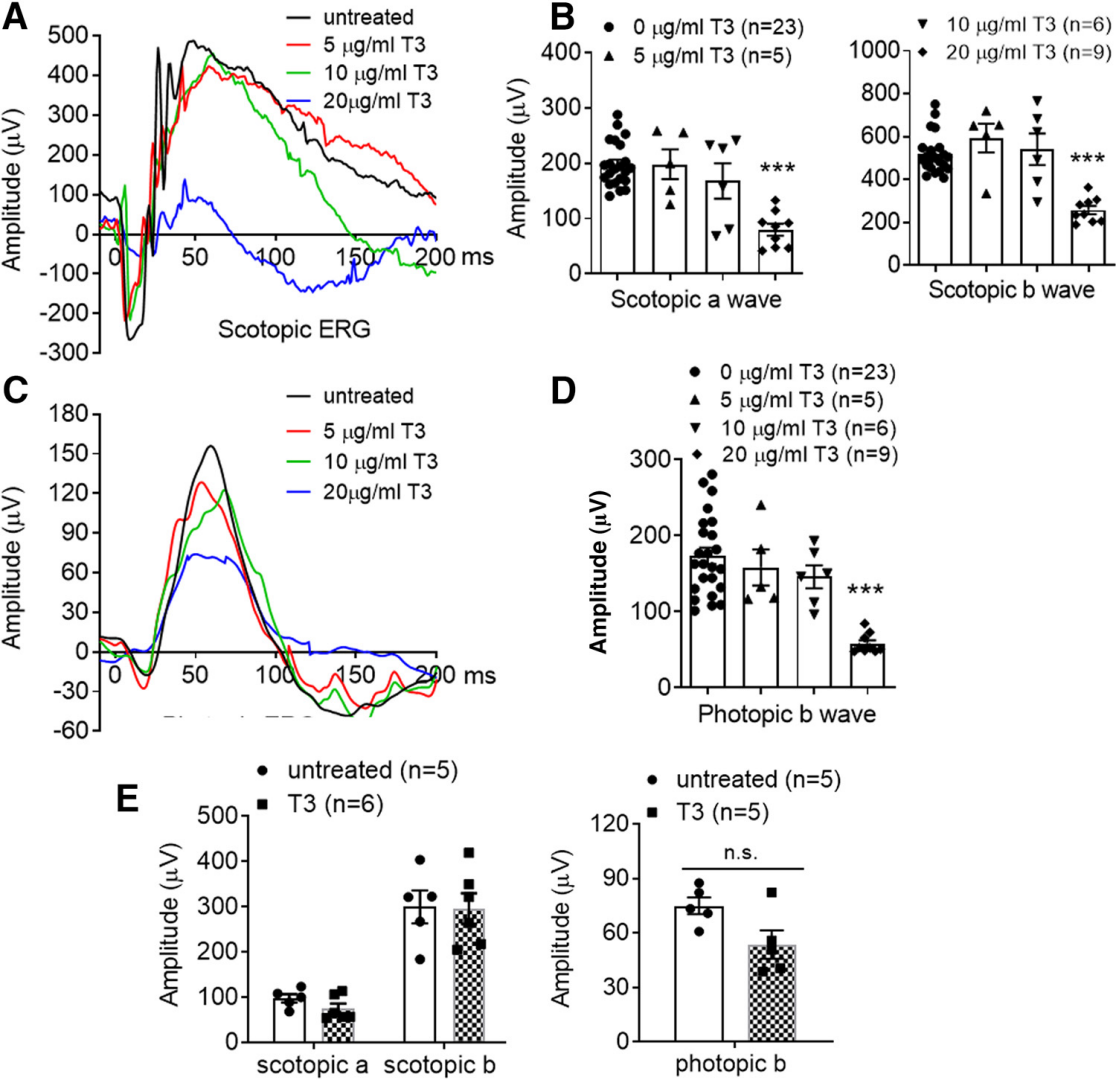
Treatment with T3 reduces retinal function. ***A–D***, C57BL/6 mice at 1 month received T3 treatment (5–20 μg/ml via drinking water) for 4 weeks and were evaluated for scotopic and photopic light responses by ERG recordings. Shown are representative scotopic waveforms and quantitative results of scotopic a/b wave amplitudes (***A***, ***B***), and representative photopic waveforms and quantitative results of photopic b wave amplitudes (***C***, ***D***). ***E***, C57BL/6 mice at 17 months received T3 treatment (20 μg/ml via drinking water) for 4 weeks and were evaluated for photoreceptor function. Shown are results of the ERG recordings. Data are represented as the mean ± SEM for 5–23 mice/group. Data were analyzed by one-way ANOVA, followed by Dunn’s multiple-comparisons test for ***B*** and ***D***, and were analyzed by unpaired Student’s *t* test/Mann–Whitney test for ***E***. **p <* *0.01, ***p <* *0.001, n.s., not statistically significant.

We first examined the effects of T3 treatment on retinal function. C57BL/6 mice at 1 month were treated with T3 at various doses (5–20 μg/ml in drinking water) for 4 weeks, and were then analyzed for retinal function using ERG. The analysis showed a significant reduction in scotopic and photopic amplitudes in mice treated with T3 at 20 μg/ml (Fig. 1*A–D*), indicating a reduction of both rod and cone function. There were no significant effects from treatments with lower doses. In a separate experiment, we examined the effects of T3 treatment on aged mice. C57BL/6 mice at 17 months were treated with T3 (20 μg/ml) for 4 weeks and were then evaluated for retinal function using ERG. The analysis showed that T3 treatment did not significantly affect the ERG responses in these mice, compared with age-matched untreated controls (Fig. 1*E*).

**Figure 2.**
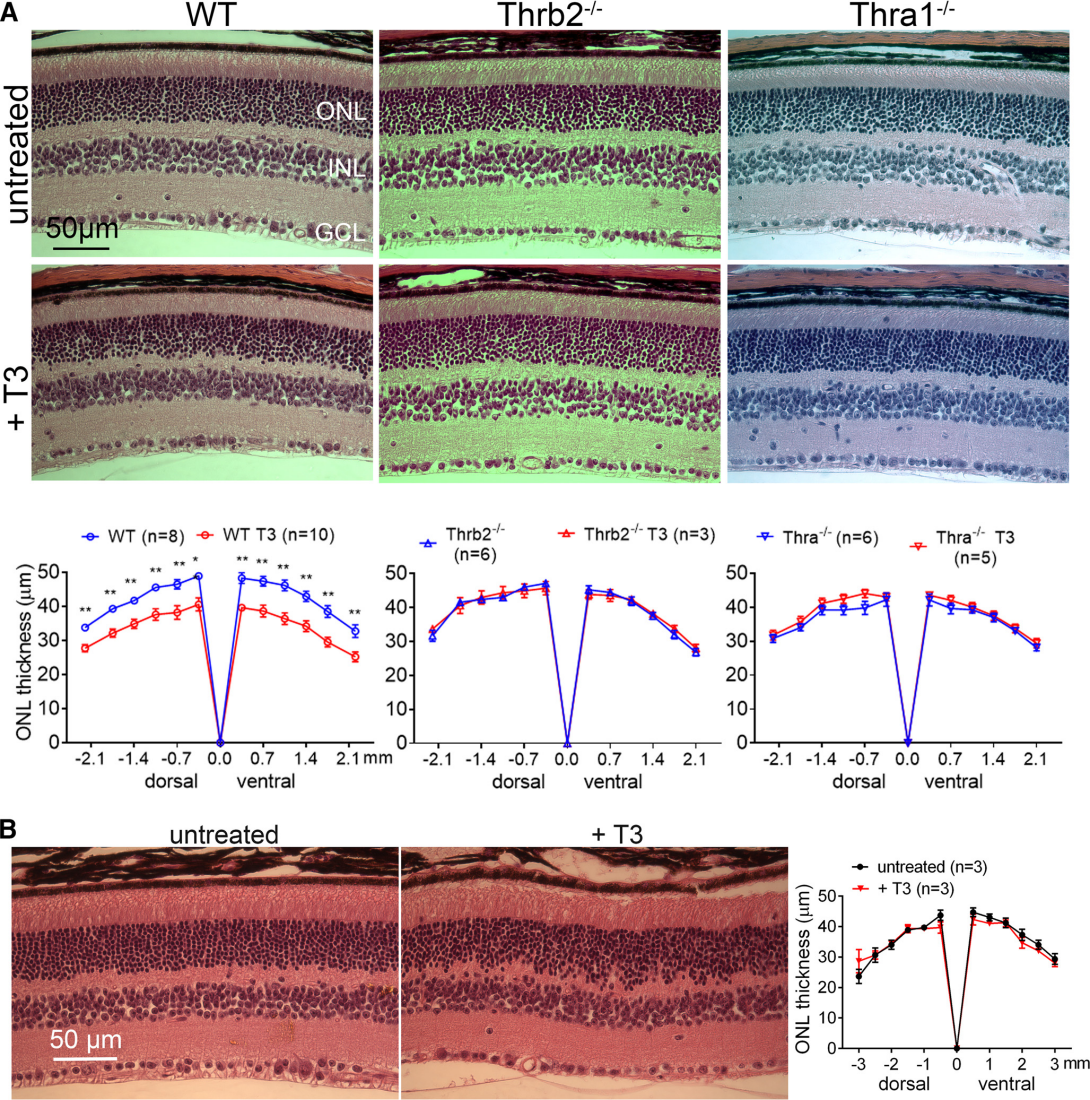
Treatment with T3 reduces ONL thickness and deletion of *Thra1* or *Thrb2* reverses this reduction. ***A***, C57BL/6, *Thra1*^−/−^, and *Thrb2*^−/−^ mice at 1 month received T3 treatment (20 μg/ml via drinking water) for 4 weeks and were evaluated for retinal morphology and ONL thickness. Shown are representative light microscopic images of H&E-stained retinal sections and corresponding quantitative analysis of ONL thickness. ***B***, C57BL/6 mice at 17 months received T3 treatment (20 μg/ml via drinking water) for 4 weeks and were evaluated for retinal integrity/ONL. Shown are representative light microscopic images of H&E-stained retinal sections and corresponding quantitative analysis of ONL thickness. ONL, outer nuclear layer; INL, Inner nuclear layer; GCL, ganglion cell layer. Data are represented as the mean ± SEM for 3–10 mice/group. Data were analyzed by unpaired Student’s *t* test/Mann–Whitney test for the two-group comparisons. *p <* *0.05, **p <* *0.01.

Morphometric analysis on H&E-stained retinal sections was conducted to evaluate the effects of T3 treatment (20 μg/ml) on rod survival/retinal integrity. The analysis showed that treatment with T3 significantly reduced the thickness of the ONL, indicating loss of rods (Fig. 2*A*, right panels). In a separate experiment, we examined the effects of T3 treatment in aged mice. C57BL/6 mice at 17 months were treated with T3 (20 μg/ml) for 4 weeks and were then evaluated for ONL thickness. Unlike that in young adult mice, there was no significant difference in ONL thickness between treated mice and age-matched, untreated controls (Fig. 2*B*). This result is consistent with the observation showing a lack of rod ERG reduction in aged mice after T3 treatment (Fig. 1*E*, left).

**Figure 3.**
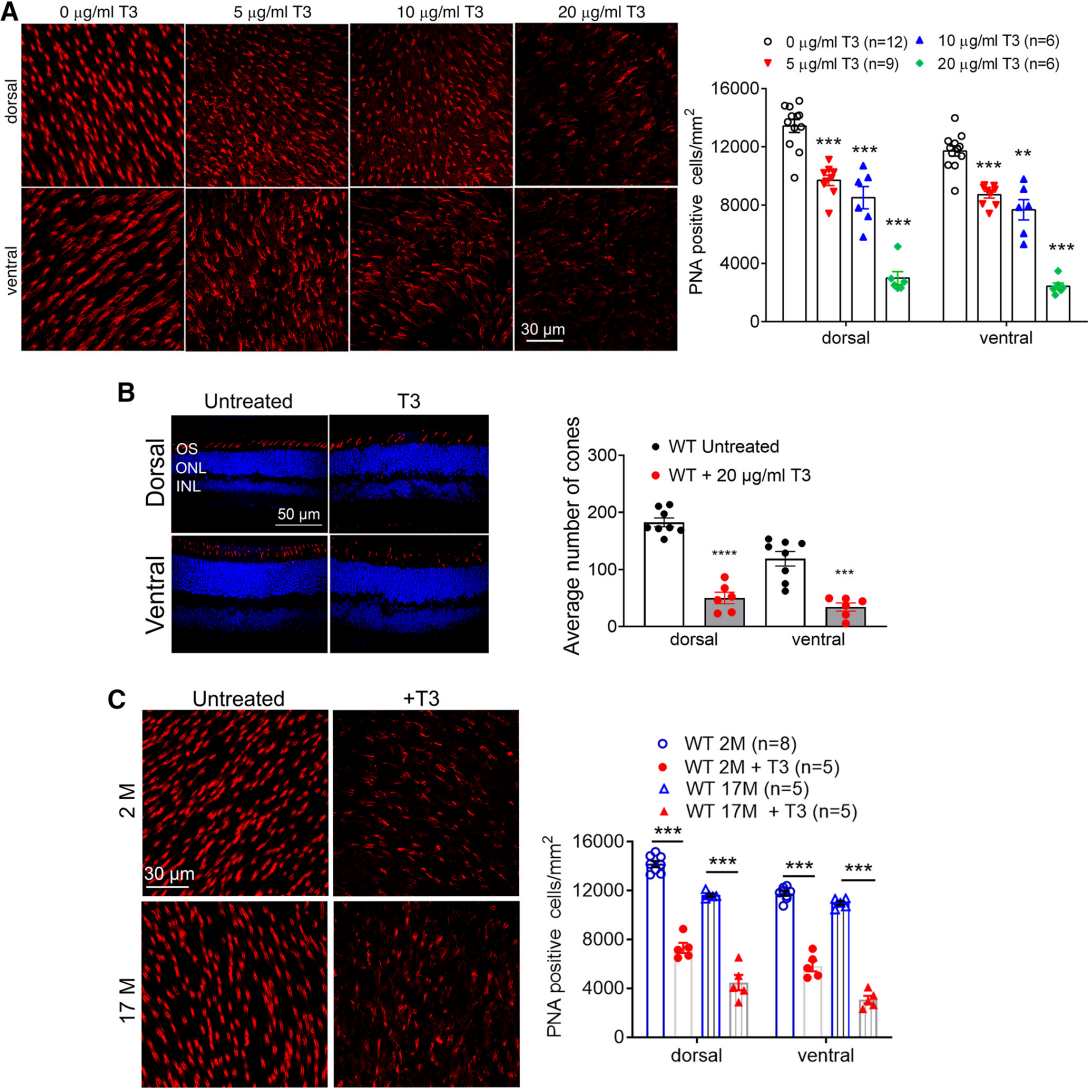
Treatment with T3 reduces cone density. ***A***, C57BL/6 mice at 1 month received T3 treatment (5–20 μg/ml via drinking water) for 4 weeks and were evaluated for cone density. Shown are representative confocal images of PNA labeling on retinal whole mounts and corresponding quantitative analysis of PNA-positive cells. ***B***, Shown are representative confocal images of immunofluorescence labeling of M-opsin on retinal sections and corresponding quantitative analysis. ***C***, C57BL/6 mice at 17 months received T3 treatment (20 μg/ml via drinking water) for 4 weeks and were evaluated for cone density. Shown are representative confocal images of PNA labeling on retinal whole mounts and corresponding quantitative analysis of PNA-positive cells. Data are represented as the mean ± SEM for 6–12 mice/group. ONL, outer nuclear layer; INL, Inner nuclear layer; GCL, ganglion cell layer. Data were analyzed by one-way ANOVA, followed by Dunn’s multiple-comparisons test for ***A***, and were analyzed by unpaired Student’s *t* test/Mann–Whitney test for ***B*** and ***C***. **p <* *0.01, ***p <* *0.001.

Immunolabeling of cone photoreceptor markers was performed to evaluate cone survival. PNA labeling of retinal whole mounts showed that T3 treatment reduced cone density in a dose-dependent manner. The reduction was observed at a dose as low as 5 μg/ml, with the lowest cone density being observed at 20 μg/ml (Fig. 3*A*). Both dorsal and ventral retinas showed a similar progression in degeneration (Fig. 3*A*). Similar findings were observed in analysis using M-opsin as a cone marker on retinal cross sections, showing reduced cone density after T3 treatment (Fig. 3*B*). In a separate experiment, we examined the effects of T3 treatment in aged mice. C57BL/6 mice at 17 months were treated with T3 (20 μg/ml) for 4 weeks and were then evaluated for cone density by PNA labeling on retinal whole mounts. The evaluation showed that T3 treatment significantly reduced cone density in aged mice compared with age-matched, untreated controls (Fig. 3*C*).

### Deletion of *Thra1* preserves rods but not cones from T3-induced degeneration

**Figure 4.**
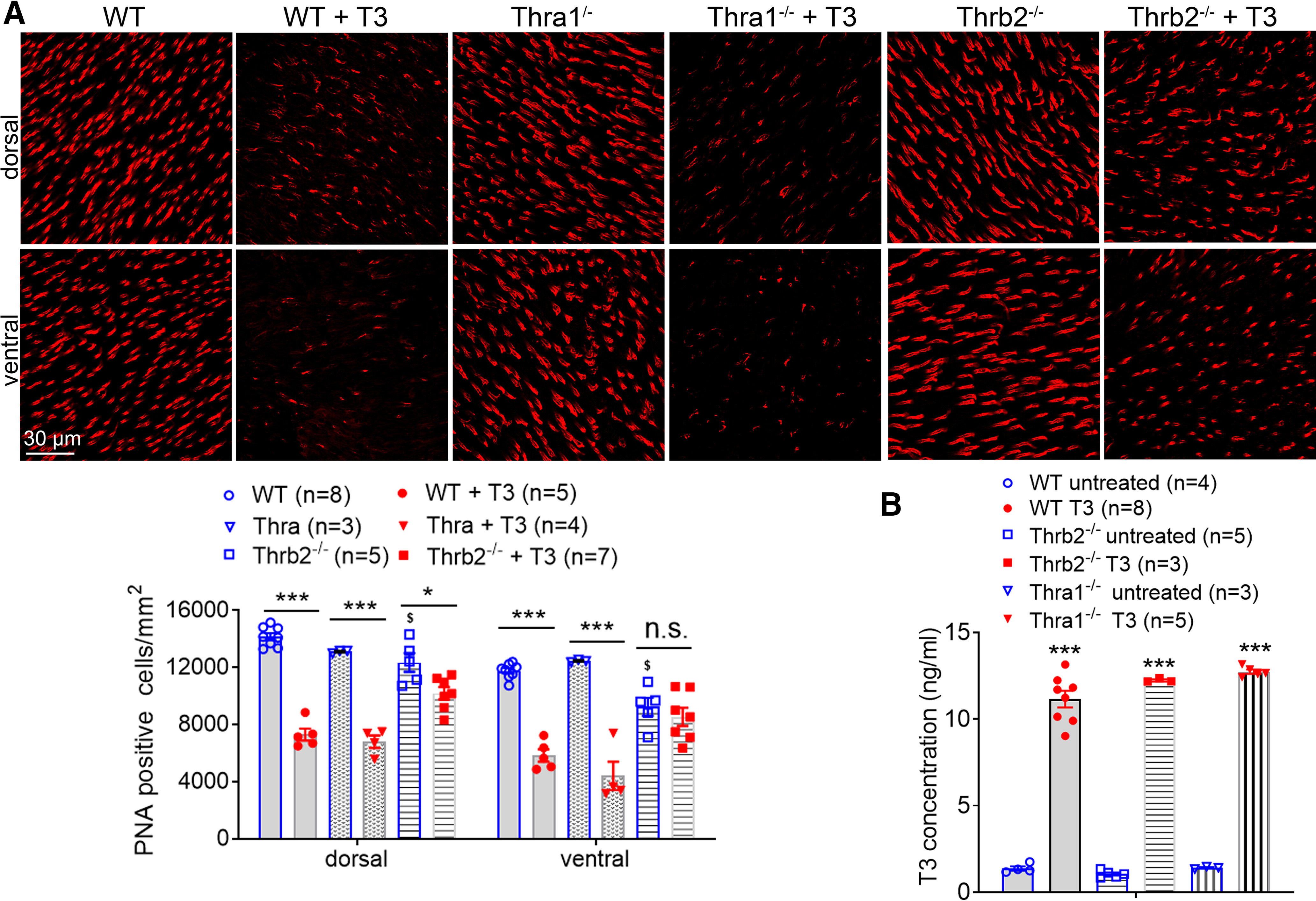
Deletion of *Thrb2* but not *Thra1* preserves cones from T3-induced degeneration. C57BL/6 (WT), *Thra1*^−/−^, and *Thrb2*^−/−^ mice at 1 month received T3 treatment (20 μg/ml via drinking water) for 4 weeks and were evaluated for cone density and serum T3 level. ***A***, Shown are representative confocal images of PNA labeling on retinal whole mounts and corresponding quantitative analysis of PNA-positive cells. ***B***, Shown are serum T3 levels analyzed by ELISA. Data are presented as the mean ± SEM for 3–8 mice/group. Data were analyzed by unpaired Student’s *t* test/Mann–Whitney test. *p <* *0.05, ***p <* *0.001; ^$^*p *<* *0.05, compared with WT.

We then examined the role of TH receptors on T3-induced photoreceptor degeneration. We first examined the contributions of *Thra1* using *Thra1*^−/−^ mice. C57BL/6 mice [wild-type (WT)] and *Thra1*^−/−^ mice at 1 month were treated with T3 (20 μg/ml) for 4 weeks, and were then analyzed for retinal integrity and cone density. We found that the deletion of *Thra1* reversed T3-induced reduction in ONL thickness (Fig. 2*A*, right panels). In contrast, deletion of *Thra1* did not prevent reduction of cone density after T3 treatment (Fig. 4*A*).

### Deletion of *Thrb2* preserves both rods and cones from T3-induced degeneration

We next examined the contribution of *Thrb2* using *Thrb2*^−/−^ mice. C57BL/6 and *Thrb2*^−/−^ mice at 1 month were treated with T3 (20 μg/ml) for 4 weeks and were then analyzed for retinal integrity and cone density. We found that the deletion of *Thrb2* reversed T3-induced reduction in ONL thickness (Fig. 2*A*, middle panels) and preserved cones from T3-induced degeneration (Fig. 4*A*). Of note, cone density in retinas of *Thrb2*^−/−^ mice was significantly lower than that in the age-matched wild-type mice and *Thra1*^−/−^ mice, and there was no difference in cone density between *Thra1*^−/−^ and wild-type mice (Fig. 4*A*). Serum T3 levels in T3-treated and control mice were examined by ELISA. Treatment with T3 increased serum T3 level by fivefold to eightfold in wild-type, *Thrb2*^−/−^, and *Thra1*^−/−^ mice, compared with their respective, untreated controls (Fig. 4*B*).

### Treatment with T3 induces photoreceptor cell death

**Figure 5.**
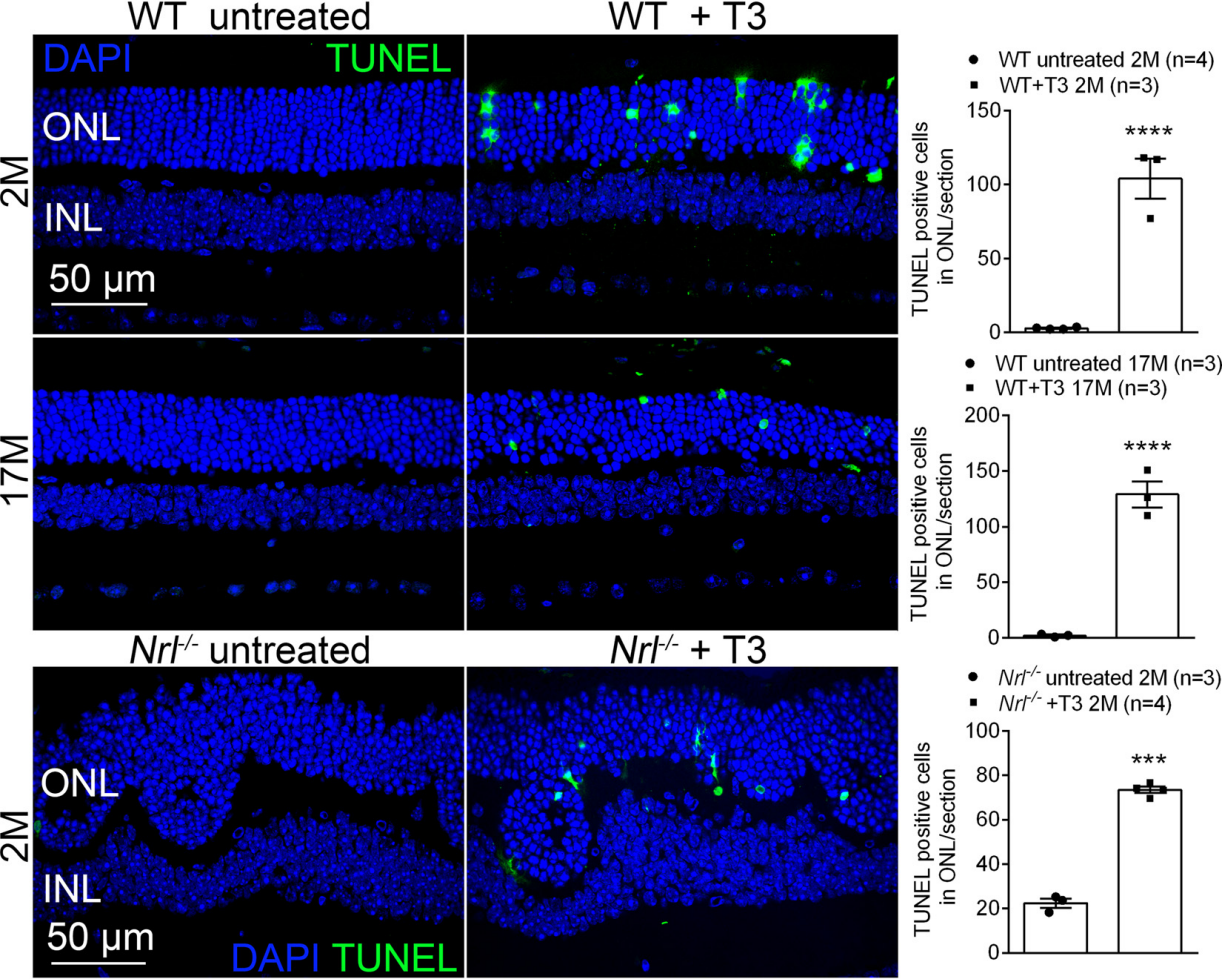
Treatment with T3 induces photoreceptor cell death. C57BL/6 mice at 1 month and 17 months and *Nrl*^−/−^ mice at 1 month received T3 treatment (20 μg/ml via drinking water) for 4 weeks and were evaluated for photoreceptor cell death. Shown are representative confocal images of TUNEL labeling on retinal sections and corresponding quantitative analysis of TUNEL-positive cells. ONL, outer nuclear layer; INL, Inner nuclear layer. Data are represented as the mean ± SEM for 3–4 mice/group. Data were analyzed by unpaired Student’s *t* test/Mann–Whitney test. ***p <* *0.001.

Photoreceptor death was evaluated by TUNEL on retinal sections. C57BL/6 mice at 1 and 17 months were treated with T3 (20 μg/ml) for 4 weeks and were then analyzed for retinal cell death. The analysis showed that T3 treatment greatly increased the number of TUNEL-positive cells in the ONL area, compared with their age-matched, untreated controls, indicating the death of photoreceptors (Fig. 5). In a separate experiment, we used *Nrl*^−/−^ mice, a model with cone-dominant retinas (Mears et al., 2001; Nikonov et al., 2005), to determine the death of cones (cones in a mammalian retina constitute only 3–5% of the total photoreceptor population). The analysis showed that T3 treatment greatly increased the number of TUNEL-positive cells in *Nrl*^−/−^ mice, indicating the death of cones (Fig. 5).

### Treatment with T3 increases expression of the genes involved in cellular necroptosis and inflammation in the retina and the involvement of TH receptors

**Figure 6.**
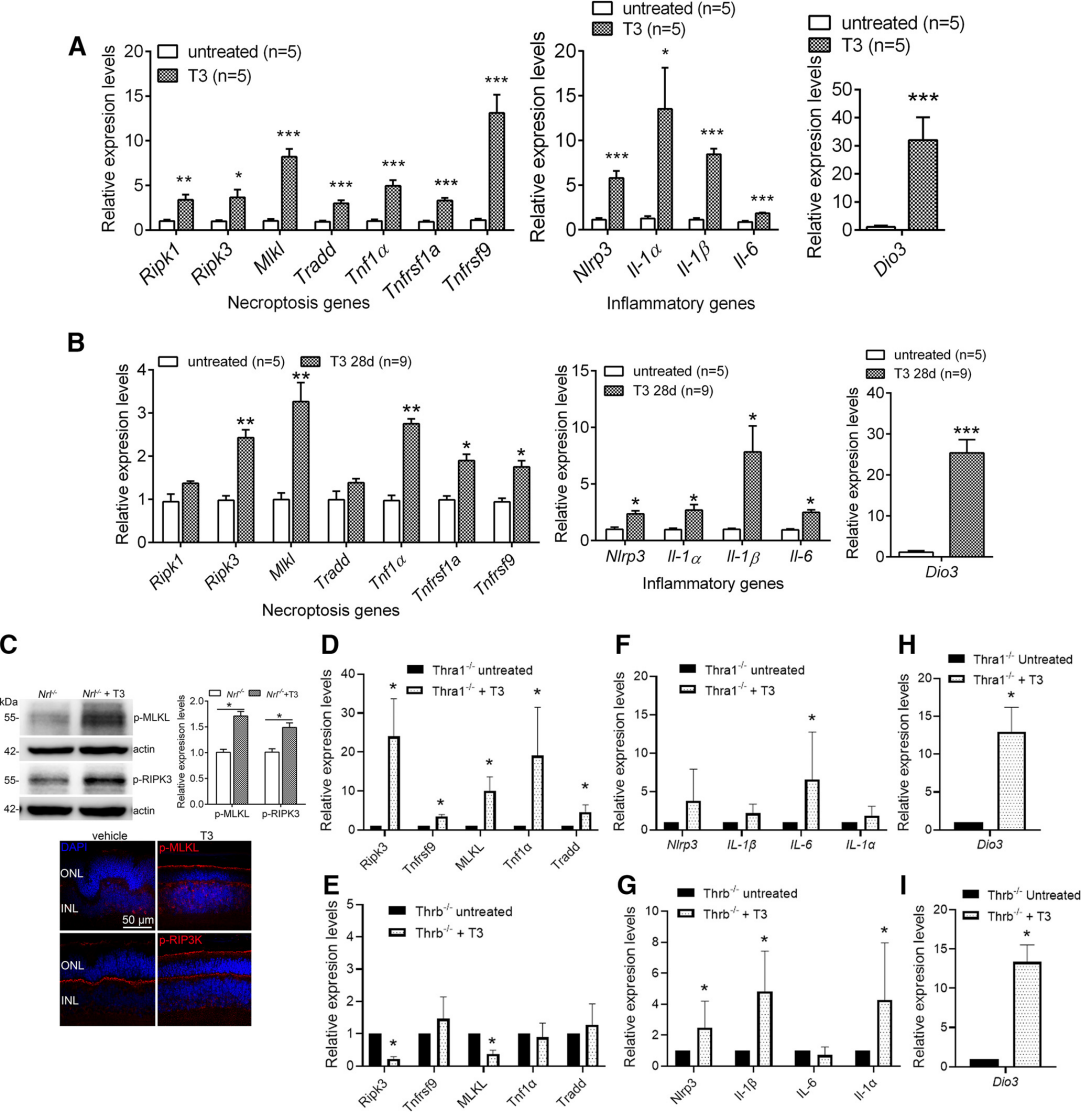
Treatment with T3 induces expression of genes involved in necroptosis and inflammation in the retina and the distinct involvement of TH receptors. ***A***, ***B***, C57BL/6 mice and *Nrl*^−/−^ mice at 1 month received T3 treatment (20 μg/ml via drinking water) for 4 weeks and were evaluated for expression levels of the genes involved in cellular stress responses and death signaling in the retina. Shown are the qRT-PCR results for expression levels of the genes involved in necroptosis and inflammatory responses in C57BL/6 (***A***) and *Nrl*^−/−^ (***B***) mice. ***C***, Shown are the results of immunoblotting with corresponding quantitative analysis and immunolabeling for phospho-MLKL and phospho-RIPK3 in *Nrl*^−/−^ mice. ***D–I***, *Thra1*^−/−^ mice and *Thrb*^−/−^ mice at 1 month received T3 treatment (20 μg/ml via drinking water) for 2 weeks and were evaluated for gene expression. Shown are the qRT-PCR results for expression levels of the genes involved in necroptosis (***D***, ***E***) and inflammatory (***F***, ***G***) responses in *Thra1*^−/−^ mice (***D***, ***F***), and *Thrb*^−/−^ mice (***E***, ***G***), and expression levels of *Dio3* (***H***, ***I***). Data are represented as the mean ± SEM of 3–5 assays using retinas prepared from 3–9 mice/group. Data were analyzed by unpaired Student’s *t* test/Mann–Whitney test. *p <* *0.05, **p <* *0.01, ***p <* *0.001.

We next examined the effects of excessive TH signaling on the expression of the genes involved in cell death/necroptosis and inflammation. C57BL/6 and *Nrl*^−/−^ mice at 1 month were treated with T3 (20 μg/ml) for 4 weeks and were then analyzed for mRNA/protein expression by qRT-PCR, immunoblotting, and immunolabeling. qRT-PCR analysis revealed that multiple genes regulating necroptosis and inflammation were significantly increased in mice that received T3 treatment, compared with their respective, untreated controls (Fig. 6*A*,*B*). The expression of *Dio3*, the gene that encodes the enzyme Dio3 which is responsible for the degradation of T3 and is stimulated by T3, was greatly upregulated, indicating increased cellular TH signaling (Fig. 6*A*,*B*). Expression levels of the necroptosis components/markers phospho-MLKL (pseudokinase mixed lineage kinase domain-like protein) and phospho-RIPK3 (receptor interacting protein kinase 3; Wu et al., 2013; Moriwaki et al., 2015) were further analyzed by immunoblotting and immunolabeling. The analyses showed increased levels of phospho-MLKL and phospho-RIPK3 in mice after T3 treatment (Fig. 6*C*). These data support the idea that excessive TH signaling induces/accelerates photoreceptor necroptosis, which may result in cell loss/degeneration.

In a separate experiment, we examined the effects of TH receptor deletion on T3-induced gene upregulation. *Thra1*^−/−^ and *Thrb*^−/−^ mice were treated with T3 (20 μg/ml, via drinking water) for 2 weeks, and were then analyzed for gene expression in the retinas. The data showed that the deletion of TH receptors selectively affects T3-induced gene expression. Among the necroptotic genes examined, the deletion of *Thra1* did not prevent T3-induced expression (Fig. 6*D*). In contrast, the deletion of *Thrb* prevented the T3-induced upregulation (Fig. 6*E*). Of note, treatment with T3 even reduced the expression of *Ripk3* and *Mlkl* in *Thrb*^−/−^ mice (Fig. 6*E*). Among the inflammatory genes examined, the deletion of *Thra1* prevented the T3-induced upregulation, except for *Il-6* (Fig. 6*F*). In contrast, the deletion of *Thrb* did not prevent the T3-induced upregulation, except for *Il-6* (Fig. 6*G*). Deletion of *Thra1* or *Thrb* did not prevent T3-induced upregulation of *Dio3* (Fig. 6*H*,*I*).

### Treatment with T3 induces photoreceptor oxidative stress/damage

**Figure 7.**
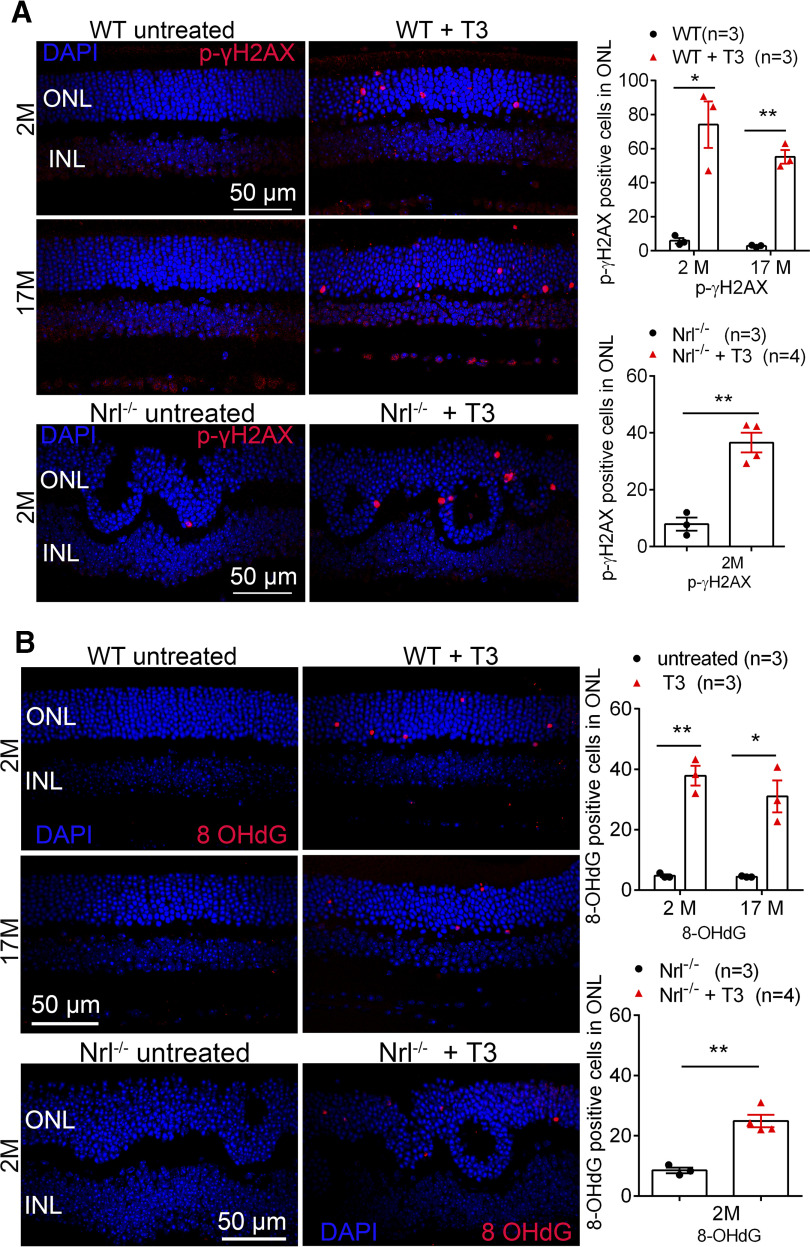
Treatment with T3 increases oxidative stress/damage in the retina. C57BL/6 mice at 1 month and 17 months, and *Nrl*^−/−^ mice at 1 month received T3 treatment (20 μg/ml via drinking water) for 4 weeks and were evaluated for oxidative stress/damage in the retina. ***A***, ***B***, Shown are representative confocal images of p-γH2AX (***A***) and 8-OHdG (***B***) labeling on retinal sections and corresponding quantitative analysis. Data are represented as the mean ± SEM for 3–4 mice/group. ONL, outer nuclear layer; INL, Inner nuclear layer. Data were analyzed by unpaired Student’s *t* test/Mann–Whitney test. *p <* *0.05, **p <* *0.01.

**Figure 8.**
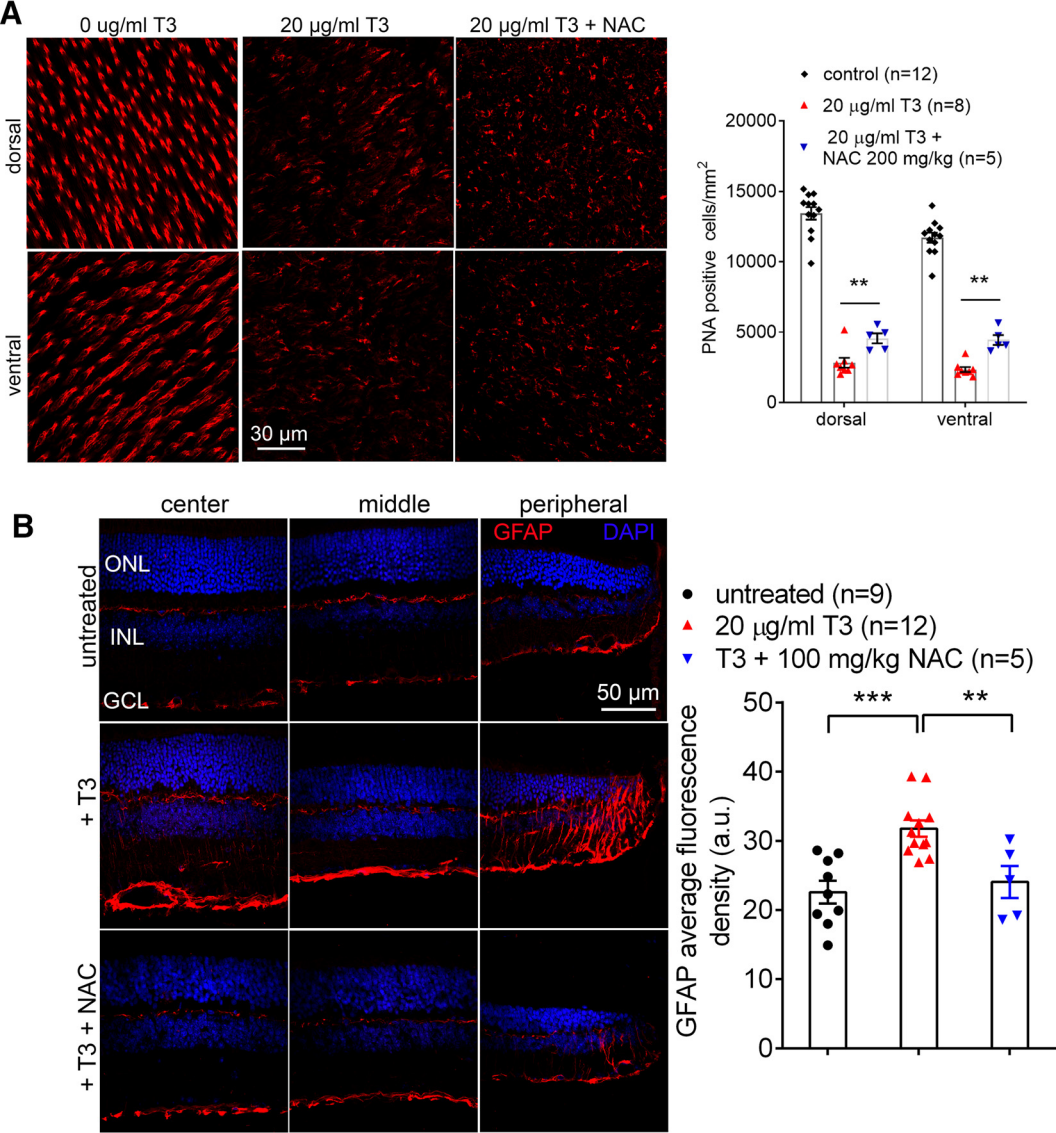
Treatment with NAC reduces T3-induced cone degeneration and inhibits Müller cell activation. C57BL/6 mice at 1 month received T3 treatment (20 μg/ml via drinking water) for 4 weeks in the absence and presence of NAC (200 mg/kg/d, i.p.) and were evaluated for cone density and Müller cell activation. ***A***, Shown are representative confocal images of PNA labeling on retinal whole mounts and corresponding quantitative analysis. ***B***, Shown are representative confocal images of GFAP labeling on retinal sections and corresponding quantitative analysis. ONL, outer nuclear layer; INL, Inner nuclear layer; GCL, ganglion cell layer. Data are represented as the mean ± SEM for 5–12 mice/group. Data were analyzed by unpaired Student’s *t* test/Mann–Whitney test for ***A*** and were analyzed by one-way ANOVA, followed by Dunn’s multiple-comparisons test for ***B***. **p <* *0.01, ***p <* *0.001.

We further evaluated the association between excessive TH signaling and oxidative stress/damage in the retina by examining the relevant markers. C57BL/6 mice at 1 and 17 months were treated with T3 (20 μg/ml) for 4 weeks and were then analyzed for oxidative stress/damage. Immunolabeling of the oxidative stress/damage markers p-γH2AX (Fig. 7*A*) and 8-OHdG (Fig. 7*B*) showed an increased number of positive cells in T3-treated mice compared with age-matched, untreated controls. *Nrl*^−/−^ mice were used as a model of cone-dominant retina to determine oxidative stress/damage of cones. The analysis showed that T3 treatment greatly increased the number of p-γH2AX-positive cells and 8-OHdG-positive cells in *Nrl*^−/−^ mice compared with age-matched, untreated controls, indicating oxidative stress/damage of cones (Fig. 7*A*,*B*). These data demonstrate that excessive TH signaling induces oxidative stress/damage in photoreceptors, possibly resulting in degeneration/cell death.

### Treatment with an antioxidant partially preserves cones and reduces retinal stress responses

The contribution of oxidative stress/damage to T3-induced cell death was evaluated using an antioxidant. NAC stimulates the biosynthesis of glutathione to combat reactive oxygen species, has been shown to protect photoreceptors in oxidative stress models (Schimel et al., 2011), and was used in this study. C57BL/6 mice were treated with T3 (20 μg/ml) for 4 weeks in the absence and presence of NAC (200 mg/kg/d, i.p.) and were analyzed for cone density at the end of the experiments. Immunolabeling of PNA on retinal whole mounts revealed that treatment with NAC partially preserved cones from T3-induced cell loss/degeneration, compared with untreated controls (Fig. 8*A*). Müller glial cells provide nourishment to retinal cells and are known to activate in response to stress by upregulating expression of GFAP. Immunolabeling of GFAP on retinal sections showed that treatment with T3 significantly increased level of GFAP/activity of Müller cells, and this elevation was nearly completely reversed by the administration of NAC (Fig. 8*B*).

## Discussion

The present work investigates the effects of excessive TH signaling on retinal function and photoreceptor viability in mice. Treatment with T3 impairs retinal function, causes rod/cone photoreceptor degeneration, and induces oxidative stress/damage, cell death, and upregulation of the genes involved in cellular stress, necroptosis, and inflammation. Deletion of *Thra1* and *Thrb2* protects photoreceptors against T3-induced cell death. These results are aligned with the findings showing photoreceptor protection by suppression of TH signaling in mouse models of retinal degeneration, demonstrating the detrimental effects of excessive TH signaling in photoreceptor degeneration.

### Excessive TH signaling induces rod and cone photoreceptor degeneration in adult mice

Although the regulation of TH signaling in cones, including development, opsin expression, and viability, has been well investigated, little is known about the effects of TH signaling in rods. This work demonstrated that excessive TH signaling induces rod degeneration in mice, manifested as reduced rod function/ERG responses and reduced ONL thickness/loss of rods. This is the first demonstration of the detrimental effects of excessive TH signaling in rods. Interestingly, excessive TH signaling did not induce a significant harmful effect in aged mice (17 months; Figs. 1*E*, 2*B*). How rods in aged mice become less sensitive to excessive TH signaling remains unclear. It might be related to the overall aging process in rods, resulting in reduced expression/activity of TH signaling components.

Excessive TH signaling has been shown to be detrimental to developing cones. Treatment with T3 or deletion of *Dio3* induced cone death in developing mice (Ng et al., 2010). The present work examined the effects of excessive TH signaling in adult mice at 1 and 17 months, and demonstrated the detrimental effects in cones, manifested as reduced cone function and cone density. Our data also suggest that cones in aged mice are more sensitive to elevated TH signaling than that in rods. The observation is in line with the clinical finding showing the correlation between elevated TH levels and increased incidence of AMD, which involves degeneration of cones following dystrophy of the retinal pigment epithelium.

### Deletion of TH receptors preserves rods and cones from T3-induced degeneration

T3 acts via TH receptors that belong to the nuclear hormone receptor superfamily and function as ligand-dependent transcription factors (Forrest et al., 2002), thereby regulating gene expression.

Two genes, *THRA* and *THRB*, encode related receptors across vertebrate species (Forrest et al., 2002; Flamant et al., 2006). Thra1 is encoded by the *Thra* gene, and the two Thrb isoform splicing variants, Thrb1 and Thrb2, are encoded by the *Thrb* gene. These receptor subtypes are broadly expressed in a variety of tissues, including the retina (Trimarchi et al., 2008; Ng et al., 2009a; Volkov et al., 2020). This work examined the involvement of TH receptors in T3-induced photoreceptor degeneration using mice with deletion of *Thra1* and *Thrb2*. Deletion of TH receptors preserved rods and cones from T3-induced degeneration (Figs. 2, 4). The profound protection from deficiency of *Thra1* and *Thrb2* supports the predominant action of T3 via its receptors. Deficiency of TH receptors leads to distinct protection in T3-induced photoreceptor degeneration. Knocking out *Thra1* preserved rods but not cones, suggesting a predominant role of Thra1 in rods but not cones in T3-induced degeneration. Knocking out *Thrb2* preserved both rods and cones, suggesting a contribution of Thrb2 in both types of photoreceptors. Nevertheless, Thrb2 has been previously shown to be expressed only in cones in the retina (Applebury et al., 2007; Jones et al., 2007; Ng et al., 2009a). The protection of rods from *Thrb2* deletion observed in this study suggests a role of Thrb2 in rods as well.

We also observed that cone density in retinas of *Thrb2*^−/−^ mice is significantly lower than that in the age-matched wild-type and *Thra1*^−/−^ mice, and that there is no difference in cone density between *Thra1*^−/−^ and wild-type mice (Fig. 4*A*). Early studies have shown that Thrb2 plays an important role in cone function in mice and that deficiency of this receptor isoform impairs cone function (Ng et al., 2001). The observation showing cone density is reduced in *Thrb2*^−/−^ mice supports a critical role of Thrb2 in cone integrity/survival. Mutations in the *THRB2* gene impair M/L cone function in humans (Weiss et al., 2012). A more detailed age-dependent change of cone survival in *Thrb2*^−/−^ mice merits further investigation. Nevertheless, cones in *Thrb2*^−/−^ mice appear resistant to TH toxicity, suggesting that Thrb2 might be the main receptor isoform for action of T3 in the mouse cones.

### T3-induced photoreceptor degeneration involves multiple cellular and molecular mechanisms

TUNEL on retinal sections revealed the death of rods and cones after T3 treatment, suggesting that the degeneration/loss of photoreceptors is attributed to cell death. Evaluation of the markers for oxidative stress/damage revealed profound oxidative stress/damage in the retina after T3 treatment. Moreover, treatment with an antioxidant partially rescued the loss of cones and reduced retinal stress responses. These data support a potential contribution of oxidative stress/damage at least in part to T3-induced cone degeneration. Nevertheless, TH signaling has been shown to contribute to oxidative stress-induced photoreceptor death. Treatment with antithyroid drugs or deletion of TH receptors suppresses retinal oxidative stress/damage and preserves photoreceptors in a NaIO_3_-induced mouse model of AMD (Ma et al., 2020, 2022), a model with oxidative stress/damage (Enzmann et al., 2006; Wang et al., 2014). In this study, we did not observe any rod protection from NAC treatment. The ONL thickness in NAC-treated mice was not different from that in mice treated with T3 only (data not shown). The lack of the rod protection or partial protection may suggest that the rod degeneration involves more complex cellular mechanisms and merits further investigation.

Necroptosis is a regulated caspase-independent cell death mechanism that resembles necrosis (Vandenabeele et al., 2010; Linkermann and Green, 2014) and is characterized by activation of the death receptors, including the tumor necrosis factor receptor superfamily (TNFRSF) and RIPK/MLKL signaling. RIPK/MLKL-mediated necroptosis has been linked to the death of photoreceptors in animal models of retinal degeneration (Murakami et al., 2012, 2015; Genini et al., 2013; Sato et al., 2013; Barabino et al., 2016; Viringipurampeer et al., 2016; Huang et al., 2018). In the present study, we examined the potential involvement of necroptosis signaling in T3-induced photoreceptor death. qRT-PCR and immunoblotting analysis showed that treatment with T3 induced expression/activation of the necroptosis component genes/proteins, suggesting an increased necroptotic signaling activity. In addition, treatment with T3 induced upregulation of several genes involved in inflammatory responses, suggesting an increased inflammatory response. Thus, TH signaling-induced photoreceptor degeneration likely involves multiple cellular and molecular mechanisms/signaling pathways. This speculation is also supported by the observation that administration of an antioxidant (NAC) did not suppress the T3-induced upregulation of the genes involved in cellular stress/death responses examined (i.e., inflammatory responses and necroptosis; data not shown). The observation suggests that the upregulation of the genes might be induced by elevated T3 signaling directly, instead of through oxidative stress/damage, the oxidative stress/damage might not be the sole factor involved in T3-induced gene upregulation, or the inhibition of oxidative stress by NAC might not be sufficient enough to inhibit the T3-induced gene upregulation.

### Distinct involvement of TH receptors in T3-induced upregulation of the genes examined

The involvement of TH receptors in T3-induced gene upregulation was examined using *Thra1*^−/−^ and *Thrb*^−/−^ mice. We found that deletion of TH receptors selectively affects T3-induced gene expression. Deletion of *Thra1* but not *Thrb* prevented the upregulation of the inflammatory genes (except for *Il-6*) examined, suggesting a primary role of Thra1 in T3-induced upregulation of these genes. Deletion of *Thrb* but not *Thra1* prevented the upregulation of the necroptotic genes examined, suggesting a primary role of Thrb in T3-induced upregulation of these genes. Interestingly, treatment with T3 even reduced the expression of *Ripk3* and *Mlkl* in *Thrb*^−/−^ mice. How T3 treatment reduces the expression of these genes in mice lacking Thrb remains unclear at this time and merits further investigation. It is worth noting that deletion of *Thra1* or *Thrb* did not prevent T3-induced upregulation of *Dio3*, the enzyme that degrades T3, suggesting a dual regulation of *Dio3* expression by both types of TH receptors.

### The potential of targeting TH signaling for photoreceptor protection

The present study demonstrates the detrimental effects of excessive TH signaling in mouse photoreceptors, supporting the view of targeting TH signaling for photoreceptor protection. This view is also favored by the following findings. (1) Suppression of TH signaling protects cones and rods in mouse models of retinal degeneration. This has been shown by treatment with antithyroid drugs (Ma et al., 2017, 2020) or by targeting intracellular TH components, including the iodothyronine deiodinases (Yang et al., 2016; Ng et al., 2017; Yang et al., 2018a, b) and TH receptor (Ma et al., 2017, 2022). The ability of the suppression of TH signaling to reduce cell death/degeneration has also been demonstrated in other neuronal and non-neuronal cells (Peeters et al., 2003; Simonides et al., 2008; Ng et al., 2009b; Bouzaffour et al., 2010; Jo et al., 2012; Dentice et al., 2014; Bhumika et al., 2015). (2) TH signaling is potentially elevated in the degenerating retinas. Previous studies have shown that degenerating retinas in mouse models of retinal degeneration show increased expression of TH receptor/Thrb2 (Ma et al., 2017) and iodothyronine deiodinases/Dio2 (Yang et al., 2016), suggesting that TH signaling activity is likely locally elevated in degenerating retinas. (3) TH signaling has been associated with human cone/retinal diseases. Population-based/patient-based studies have shown strong evidence for the association between elevated TH level in the circulation/hyperthyroidism and increased incidence of AMD (atrophic/dry AMD; Age-Related Eye Disease Study Research Group, 2000; Chaker et al., 2015; Gopinath et al., 2016; Chatziralli et al., 2017; Lin et al., 2018; Abdelkader and Abass, 2019; Farvardin et al., 2021; Xu et al., 2021; Hung et al., 2022; Li et al., 2022). Optical coherence tomography evaluation shows macular thinning in patients with thyroid-associated ophthalmopathy (Sayın et al., 2016; Blum Meirovitch et al., 2017). Thus, the use of Dio2 inhibitors or TH receptor antagonist locally in the retina (Yang et al., 2016) might be worth testing for photoreceptor protection.

In summary, the present work demonstrates that excessive TH signaling is harmful to photoreceptors, leading to photoreceptor degeneration and impairment of retinal function. The TH signaling-induced photoreceptor degeneration likely involves multiple mechanisms, including oxidative stress/damage, necroptosis, and inflammation. Deletion of *Thra1* and *Thrb2* leads to protection against T3-induced photoreceptor degeneration. The findings from this study support a role of TH signaling in the progression of photoreceptor death in retinal degeneration. Along with the experimental findings showing the protection of suppression of TH signaling in mouse models of retinal degeneration and the clinical findings showing the link between high serum TH levels/hyperthyroidism and increased incidence of AMD, the present work supports the view that inhibition of TH signaling might be a valuable strategy for photoreceptor protection in retinal degeneration.

## Synthesis

Reviewing Editor: David Schoppik, New York University Decisions are customarily a result of the Reviewing Editor and the peer reviewers coming together and discussing their recommendations until a consensus is reached. When revisions are invited, a fact-based synthesis statement explaining their decision and outlining what is needed to prepare a revision will be listed below. The following reviewer(s) agreed to reveal their identity: NONE. Note: If this manuscript was transferred from JNeurosci and a decision was made to accept the manuscript without peer review, a brief statement to this effect will instead be what is listed below.

We agreed that the current work is in line with previous publications and makes a compelling connection between TH toxicity and photoreceptor cell death in adult tissue, apart from a role that TH has in development of the retina, and that the work is important for understanding the role of TH in degenerative diseases and suggesting strategies for protecting photoreceptors.

Major:

1. The quantification of genes involved in cellular necroptosis and inflammation with T3 treatment (Figure 8) would be even further bolstered with quantification with the NAC treatment and/or in the Thra1 and Thrb2 null mutants as well.

2. As for statistical analysis, it is recommended that non-parametric tests are used unless the use of parametric tests can be justified. Similarly, the manuscript needs details about post-hoc testing. This manuscript is an ideal candidate for estimation statistics, and we thought it would be considerably stronger for their use. see https://www.claridgechang.net/blog/estimation-stats-launches for a good starting point and toolboxes to make this easier.

Specific comments:

Figure 3: The opsin staining should be quantified as dorsal and ventral subsets, since every other graph is similarly quantified, and because the representative image of TH treatment suggests that there might be differences.

Figure 4. Statistical comparison between Thrb2-/- + T3 and WT+T3 and between Thra -/- + T3 and WT +T3. The symbols are sometimes difficult to discern from each other. The authors might consider using different colors for the bars or data points to better distinguish the 4 categories that are compared.

Figure 4: The presentation of the graphs in this figure is puzzling. Since the Thra1 and Thrb2 mutants use the same WT numbers as their controls, they can be consolidated into a dorsal subset and a ventral subset. That would highlight two details that need to be addressed. First, the Thr2b -/- mouse has fewer cones in the ventral domain (and is that a signficiant difference), and second, that those cones seem very resistant to TH toxicity. As the Thr2b -/- mouse is a developmental model, some discussion should be made about what might be different in this retina, that it is obviously different from the WT and Thr1a -/- retina.

Figure 8: This figure can be combined with Figure 5, as a description of what TH toxicity activates in the retina. Or consider the possibility of including the quantification across rescue conditions (see major comment).

Minor suggestions

Confusing language, underlined text could be an addition that clarifies:

Top of page 13: “Treatment with T3 increased serum T3 level by 5-8 folds in mutant and WT animals, compared with untreated controls (Fig. 4B).

Duplicates in the References section

In the Discussion section “Potential of Targeting TH receptors preserves...” p18

There are four sentences that start the exact same way-leading to some confusion over the point that is being made this section can be clarified by varying the language. Also, it is not clear what is meant by a genomic mechanism:

“Deletion of TH receptors preserved rods and cones from T3-induced degeneration (see Figs 2, 4). The profound protection from deletion of Thra1 and Thrb2 supports the predominant action of T3 via its receptors/genomic mechanism. Deletion of TH receptor shows distinct protection in T3-induced photoreceptor degeneration. Deletion of Thra1 preserved rods but not cones, suggesting a predominant role of Thra1 in rods but not cones in T3-induced degeneration. Deletion of Thrb2 preserved both rods and cones, suggesting a contribution of Thrb2 in both types of photoreceptors.”
